# Assessment of functional feeding groups (FFG) structure of aquatic insects in North- western Rif - Morocco

**DOI:** 10.3897/BDJ.11.e104218

**Published:** 2023-06-14

**Authors:** Sara El Yaagoubi, Majida El Alami, Rihab Harrak, Ahlame Azmizem, Mohamed Ikssi, Mohammed Reda Aoulad Mansour

**Affiliations:** 1 Laboratoire Ecologie, Systématique, Conservation de la Biodiversité, LESCB URL-CNRST N°18, FS, Abdelmalek Essaadi University, Tétouan 93000, Morocco Laboratoire Ecologie, Systématique, Conservation de la Biodiversité, LESCB URL-CNRST N°18, FS, Abdelmalek Essaadi University Tétouan 93000 Morocco; 2 Chef de la Division du Domaine Public Hydraulique - Agence du Bassin Hydraulique du Loukkos, Tetouan 93000, Morocco Chef de la Division du Domaine Public Hydraulique - Agence du Bassin Hydraulique du Loukkos Tetouan 93000 Morocco

**Keywords:** Aquatic insects, Collector-gatherers, Functional feeding groups, Rif, physical chemical variables

## Abstract

The involvement of trait-based approaches is crucial for understanding spatial patterns, energy flow and matter transfer in running water systems, which requires consistent knowledge of the functional structures of aquatic communities, with the advantage of combining physical properties and behavioral mechanisms of food acquisition rather than the taxonomic group. The present study indicated how functional feeding groups may be used as a proxy for classical taxonomic evaluation, as well as the potential interest in incorporating them as indicators of anthropogenic stressors. The composition and abundance of the functional feeding groups of aquatic insects were examined from September 2021 to August 2022 along the Western Rif Region.

Benthic samples were collected from nine sampling points in the studied area using a Surber sampler with a mesh size of 500 µm and a diameter of 20*20 cm. The stations included in this work were chosen for their accessibility as well as their position on the hydrographic systems. The abundance of sampled aquatic organisms in the whole study area revealed 5,342 individuals belonging to 60 families and seven orders of aquatic insects, classified into five feeding functional groups. In terms of abundance, Collector-gatherers (Ephemeroptera and Diptera) were the most abundant trophic group at most of the sites, with a proportion of 38.47%. Predators (Coleoptera, Hemiptera and Odonata) were the second group at all sites, followed by Collector-filters, accounting for 39.53%, 28.14% and 22.37% respectively, while Scarpers and Shredders had the lowest representation across all sites with 4.16%. The high number of registered Collectors could be related to their ability to feed on a diverse range of food items compared to the remaining trophic guilds. According to the Canonical Correspondence Analysis results, physicochemical (i.e. T, pH, BOD_5_, Cl- and NO_3_-) and hydromorphological (i.e. current velocity and depth) variables were amongst the key predictors of shaping the functional structure of aquatic biota during this investigation. It is highly recommended to carry out suitable measures to largely attenuate anthropogenic pressures in order to preserve the integrity of freshwater bodies and their biota.

## Introduction

The Mediterranean freshwater ecosystems have been classified as one of the most threatened aquatic entities by climate change, which affects water temperatures and supplies, leading to a progressive shift in flow regime (Döll and Zhang 2010, Knouft and Ficklin 2017). Moreover, Mediterranean streams undergo substantial hydrological alterations compared with their temperate regions. This is particularly true in North African semi-arid and arid aquatic habitats, where freshwater organisms are frequently subjected to extreme floods and drought events (Gasith and Resh 1999, Benzina et al. 2021). These changes in the local environment favor some typical trade-offs, resulting in broad alterations in the functional and taxonomic structure of the aquatic biota (Durance and Ormerod 2007).

Amongst the various aquatic organisms found in streams or rivers, benthic macroinvertebrates have unique functional forms, based on the physical or chemical conditions. As a result, benthic organisms have been largely used as bioindicators for assessing water quality and the health of aquatic habitats, based on these properties (Rosenberg et al. 1986, Smith et al. 1999, Barman and Gupta 2015, Kim and Kong 2019). Therefore, aquatic insects present an intermediate trophic level consumer and are indispensable in channeling the trophic web from bottom-up and top-down directions (Wallace and Webster 1996, Doong et al. 2021). They also shift their biological attributes and functional traits in response to stressors caused by various pressures and external drivers (Ansah et al. 2012). In terms of assessing the water quality of streams, biological monitoring is found to be more effective than the classical physicochemical-based approach (Merritt et al. 1996, Gebler et al. 2014, Addo-Bediako 2021). Indeed, the assemblage of local communities is visualised in the context of the river habitat templet as a process in which multiple habitat filters act hierarchically, selecting organisms with a set of biological traits that allow them to survive, grow and reproduce under constraining conditions (Poff and Ward 1990, Townsend and Hildrew 1994, Statzner et al. 2001).

The biological trait-based approach has been shown to be particularly effective in describing functional changes in biological communities exposed to environmental variability (Statzner et al. 2001, Horrigan and Baird 2008, Culp et al. 2011) and anthropogenic disturbances (Dolédec and Statzner 2008, Feio and Dolédec 2012). Otherwise, the functional approach appears to be less sensitive to seasonal variability, sampling effort (Charvet et al. 1998, Bady et al. 2005), taxonomic resolution level (Dolédec et al. 2000, Gayraud et al. 2003 and large-scale spatial taxonomic variability (Statzner et al. 2001, Bonada et al. 2006). Recently, a functional approach, based on macroinvertebrate functional feeding groups (FFGs), has emerged to assess the ecological integrity of aquatic habitats. The benthic fauna is considered as an indicator of ecosystem attributes (Merritt et al. 2002, Fierro et al. 2017, Dufour et al. 2019, Li et al. 2019, Edegbene et al. 2021); using a food acquisition classification system based on behavioral processes (Cummins 1975, Ramírez and Gutiérrez-Fonseca 2014) and organic resource utilisation modes (Simberloff and Dayan 1991). Thus, some functional groups, like Shredders and Scrapers are designed to be more susceptible to environmental changes, whereas Collector-gatherers and Collector-filterers are considered as pollution-resistant groups, which may affect the availability of particular food sources (Barbour et al. 1996). The functional feeding group approach is considered to fit the characterization of environmental conditions (Vannote et al. 1980, Merritt and Cummins 1996, Cummins et al. 2005, Cummins et al. 2008, Mishra and Nautiyal 2013, Cummins 2016), with the implication of abundance ratios of different categories as surrogates for ecosystem parameters to assess the ecological integrity of aquatic biota and freshwater bodies. For instance, the ratio of Scrapers plus Collector-filters to Shredders plus Collector-gatherers was used to calculate channel stability (Merritt et al. 2017). The top-down predator control was estimated as predator-to-prey ratio. Furthermore, hydrological and biological changes can interrupt the flow of allochthonous basal supplies into streams, impacting freshwater taxa's food supply. For instance, a shift in land use might have a substantial impact on the basal resources that sustain the survival of benthic macroinvertebrates, such as Grazers and Shredders (Miserendino and Masi 2010, Mangadze et al. 2019, Vilenica et al. 2020).

The influence of disturbances on the distribution and abundance of functional feeding groups in habitats was largely explored in previous studies, by reflecting the state of the habitat and water quality (Abdul and Rawi 2019), the FFGs approach has been effectively applied to different aquatic ecosystems in Latin America (Cummins et al. 2005, Gonçalves and De Menezes 2011, Cortés-Guzmán et al. 2021), Europe and Africa (Paunović et al. 2006, Moyo and Richoux 2017, Mangadze et al. 2019, Addo-Bediako 2021, Edegbene et al. 2022) and Morocco (Nahli et al. 2023). The last work and the given study present a major contribution in terms of assessing the functional structure of aquatic communities as an innovative biomonitoring approach in Morocco . Concurrently, the intensity and magnitude of anthropogenic pressures have reached their tolerance threshold in some sections of the Rif region, which is becoming more notable during the summer season, when resource requirements are greater and natural contributions and inputs remain minimal (Errochdi et al. 2012).

In this study, we evaluated the impact of anthropogenic activities on the functional feeding groups of aquatic insects in the Mediterranean northern Moroccan rivers. This is the first study of its kind within the studied area, where streams are presumably subjected to various disturbances.

## Materials and Methods

### Study area

**Figure 1.**
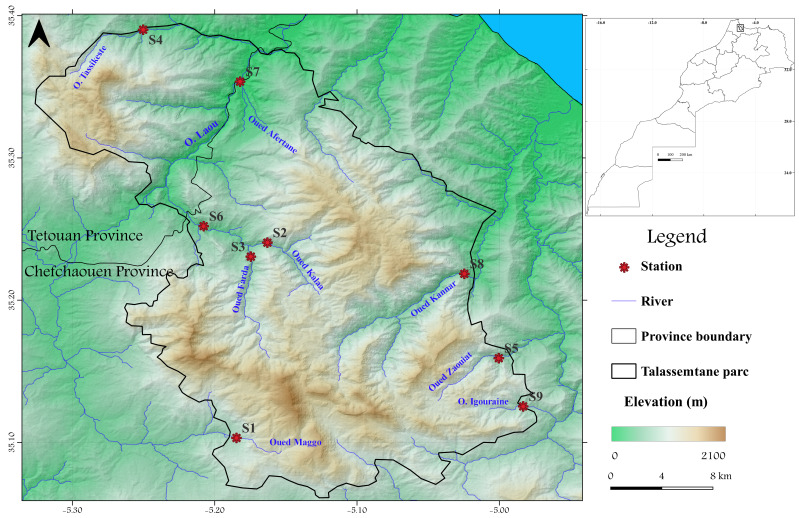
Location map of the study area and sampling sites.

The study area is a part of the Rif Region. Morocco's lone mountainous chain emerges from the alpine orogeny. It is located in the northernmost portion of the country and lies between northern latitudes 34º23' and 35º20' and western longitudes 5º13' and 5º11', respectively (Fig. 1). The Rif has a Mediterranean climate with two distinct seasons: hot and dry summers and rainy and wet winters. The sampling processing was scheduled to take advantage of the meteorological and hydrological characteristics of each site (rainfall, flood period etc.), while the stations retained for this work were chosen, based on their accessibility as well as their distribution on the hydrographic network to cover the different habitats represented in this region. Based on these criteria, nine stations were selected from three hydrographic networks: seven stations belonging to the Laou catchment, one station from the Kannar sub-catchment and one station belonging to the Bouhia sub-catchment. Thus, the Laou watershed is a Mediterranean basin in Morocco's north-western Provinces of Tetouan and Chefchaouen. It is located in the heart of the Rif chain; Laou is a tiny basin with an approximate total size of 930 km² (El Alami and Dakki 1998, Errochdi et al. 2012) extending from high altitudinal woods to agricultural and urban lands at lower elevations. The examined streams have a Mediterranean hydrological regime with high water in the late winter and early spring and low water in the summer. Their hydrological regimes are highly erratic, with severe summer droughts and intense floods in the winter significantly impacting flow fluctuations.

### Sampling, identification and FFG classification

This study was carried out at nine locations where human-induced changes in land use and source catchment provide an appropriate setting for investigating the functional responses of aquatic communities. Aquatic insects were sampled using a Surber net (20 x 20 cm), which was used to sample riffles by dislodging and removing all organisms from each rocky substrate. The collected fauna was preserved directly in 96% ethanol, after being cleaned and elutriated. All specimens were sorted and identified at the family level using the identification key of Tachet et al. (2010). The allocation of individuals to their corresponding functional groups was based on the available determination keys of Merritt and Cummins (1996) and Tachet et al. (2010). In this study, the FFG categories employed were Collector-gatherer (CG), Collector-filters (CF), Scrapers (Sc), Shredders (Sh) and Predators (P) (Suppl. material 1). The contribution percentage of each FFG to the various communities was calculated at all the examined sites.

### Physicochemical and hydromorphological parameters

Physical and chemical parameters, such as water temperature, pH, dissolved oxygen (DO), total dissolved solids (TDS), salinity and electrical conductivity (EC), were measured seasonally with a Multi-probe meter for each sampling point. Hydraulic parameters, such as velocity, river width and depth, were measured in situ (with three replicates) using a tape measure. Before further analysis, water samples were collected in 1000 ml polyethylene bottles and kept at ± 4°C. Plastic bottles of water were delivered to the Loukkos Hydraulic Basin Agency laboratory in Tetouan (ABHL, Tetouan) within 24 hours of sampling for the examination of total suspended solids (MES), five days biochemical oxygen demand (DBO_5_) and chemical oxygen demand (COD). Water samples were subjected to quantification of nutrient content (NO_2_- and NO_3_-) and complexometric determination of calcium and chloride. The mean and standard deviations of each of the measured parameters were calculated (Suppl. material 2).

### Functional composition of aquatic insects

Using the criteria of Merritt and Cummins (1996), aquatic insects were categorized into various functional feeding groups: Shredders (Sh), Collector-gatherers (CG), Collector-filters (CF), Scrapers (Sc) and Predators (P).

### Functional feeding group ratios used as indicators of stream ecosystem attributes

The FFG ratios are also used as indicators of stream ecological attributes. Table 1 derived from Cummins et al. (2005) represents the calculated ratios with their general criteria ratio levels. The ratio of Scrapers to (Shredders + total collectors [Collector-filters + Collector-gatherers]) was used to calculate the balance between autotrophy and heterotrophy (Production/Respiration) index; the ratio of Shredders to total collectors (Collector-filters + Collector-gatherers) was used to calculate the linkage between riparian inputs and stream food webs (CPOM/FPOM).

### Data Analyses

The mean and standard deviations (SD ± mean) were calculated for each abiotic variable at each sampling site. A principal component analysis (PCA) with a Varimax rotation was carried out on an environmental data matrix consisting of nine sampling sites and twelve physicochemical and hydrological parameters to determine the river typology. Hierarchical clustering analysis was conducted to assemble groups according to a criterion of similarity defined in advance, which will be expressed in the form of a matrix of distances. In a simplified way, this method seeks to minimize intraclass inertia in order to obtain the most homogeneous classes. The relationship between FFGs and environmental variables was described using Canonical Correspondence Analysis and, as its name indicates, is based on correlations and the presence or absence of a linear relationship between variables in different sets or groups. The statistical analyses were performed using *Xlstat 2022* software.

## Results

### Physicochemical and hydromorphological parameters

The average values of the respective physicochemical and hydromorphological variables for each site are shown in Suppl. material 2. At S3, the mean velocity was 0.61 m/s and at S9 it was 0.88 m/s. S2 (0.48 m) had the greatest mean water depth, while S5 had the least mean depth (0.09 m). The width of the examined locations increased gradually from upstream to downstream sites, ranging from 2.43 m at S9 to 18.96 m at S8. The mean pH values at the sites ranged from 7.20 at S6 to 7.86 at S1 and the mean temperature ranged from 14.1°C at S3 to 19.7°C at S7.

The mean of dissolved oxygen ranged from 7.27 mg/l at S16 to 9.01 mg/l at S5 and S8, respectively. The mean electrical conductivity ranged from 437 μS/cm^2^ at S5 to 654.5 μS/cm^2^ at S8. The mean TDS ranged from 236.75 ppm at S1 to 330 ppm at S8 and the average salinity values ranged from 0.17 psu at S6 to 0.27 psu at S8. The mean BOD_5_ concentrations range from 34.35 mg/l in S3 to 81.75 mg/l in S5. Station S5 had the largest nitrate concentration with 0.55 mg/l, while S8 and S7 had the lowest nitrate scores of 0.10 mg/l and 0.12 mg/l, respectively, whereas nitrite concentrations were almost lower than the detection level at the majority of sampled sites. S3 had the highest mean calcium content, whereas S8 and S9 had the lowest scores (Suppl. material 2).

### Proportion and distribution of functional feeding groups

A total of 5,342 specimens were collected from the following orders of aquatic insects: Ephemeroptera (1,741), Trichoptera (1,150), Diptera (895), Coleoptera (667), Hemiptera (378), Odonata (380) and Plecoptera (131). The aquatic insects obtained from the nine stations were listed as Collector-gatherers (n = 2,109), Predators (n = 1,503), Collector-filters (n = 1,195), Scrapers (n = 222) and Shredders (n = 222), Collector-gatherers/Scrapers (n = 75) and Predators/Scarpers (n = 16).

**Figure 2.**
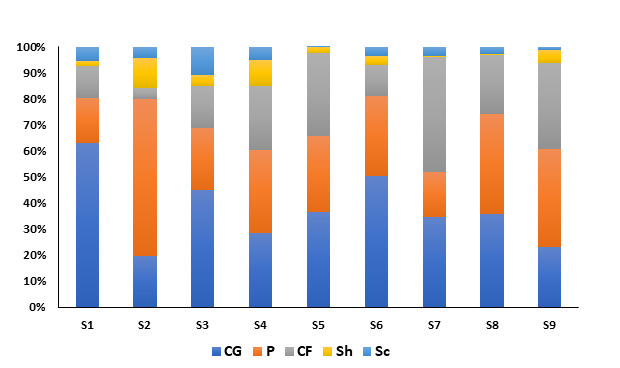
Average relative abundances (in %) of FFGs at the sampling sites (**CG**. Collector-gatherers; **CF**. Collector-filters; **Sh**. Shredders; **Sc**. Scrapers; **P**. Predators).

Collectors-gatherers were the most common category in the entire study area with an important abundance in S1 and S6. Predators were the second most common group amongst the sampled sites, with a high proportion in S2, followed by Shredders and Scrapers with a comparable abundance. Meanwhile, Table 2 shows the abundance of the main functional feeding groups at their respective sites. During all seasons, Collector-gatherers and Collector-filters were numerically dominant in the selected sites, accounting for 39.47% of the total assemblage, followed by Predators (28.14%) and Collector-filters (22.37%). The two groupings of P/Sc and CG/Sc were extremely low (Fig. 2). The average relative abundance of Collector-gatherers decreased in S2, favoring the occurrence of Predators and Shredders. The relative abundance of Scrapers decreased drastically at S5 in favor of Collector-gatherers and Collector-filters. The proportion of Collector-gatherers increased downstream, with S1 representing the highest proportion. Predators were numerically well-represented in the upstream rivers, with less or almost equal proportions in the inundated and downstream sites.

**Figure 3.**
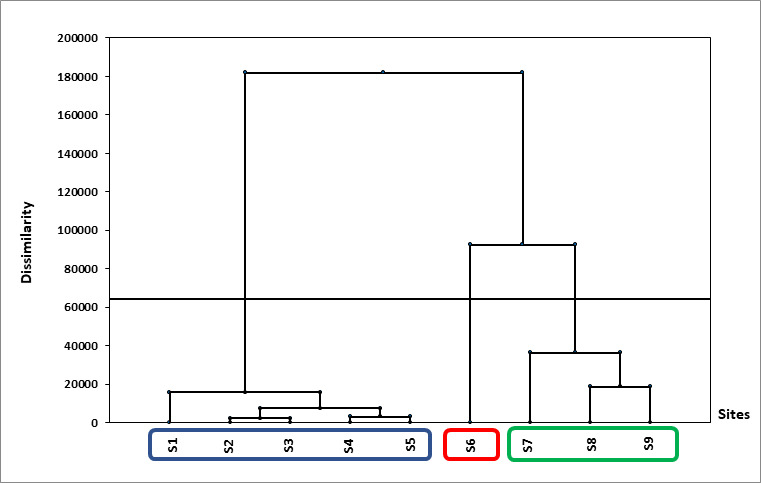
Hierarchical Clustering Analysis of studied stations.

A Hierarchical cluster analysis of aquatic communities was performed between the selected sites, revealing three distinctive groups. The number of segmented observations was the number of vertical lines that were intersected by the line drawn using the threshold. Cluster I contained the following sites: S1, S2, S3, S4 and S5, while Cluster II included only S6, whereas Cluster III comprised S7, S8 and S9 (Fig. 3). Cluster I included sites with a high magnitude of anthropogenic disturbances as well as a high abundance of tolerant taxa. Cluster III incorporated sites with higher conductivity and temperatures, but considerable spatial heterogeneity (diverse microhabitats).

### Interaction between functional structure of aquatic insects and environmental factors

**Figure 4.**
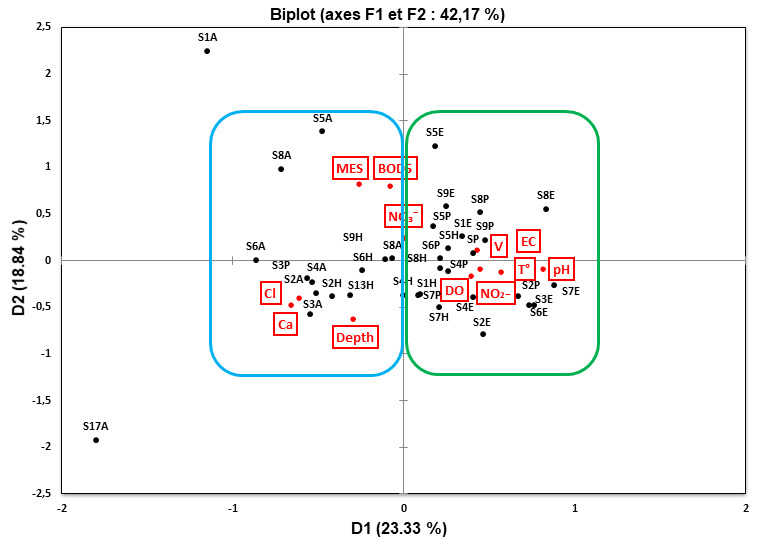
PCA biplot after Varimax rotation carried out between sites and physicochemical data.

The PCA results on the physicochemical data revealed that axes 1 and 2 (D1 and D2) explained 42.17% of the ordination of environmental predictors (Fig. 4). As a result, the stations were divided into two groups on the factorial plane D1*D2; the first group consisted of the following sites (S1, S5, S8 and S9); they provided an upper temperature, a high suspended matter concentration and an increased nutrient concentration. The second group included sites located midstream and downstream (S2, S3, S4, S6 and S7). The sites S5, S6, S8 and S9 were dispersed along axis I, which accounted for 23.33% of the total variability; the aforementioned locations were positively correlated with velocity, EC and DO and negatively related to Cl⁻ and Ca^2+^. Along axis II S2, S3, S4 and S6, explaining a further 18.84% of environmental variability, those sites were positively associated with MES and BOD_5_ and velocity and negatively related to water depth.

### Attributes of aquatic ecosystem

The use of the P/R ratio revealed that all stations were heterotrophic (P/R < 0.75), except S7, which has a slightly higher P/R ratio (P/R = 0.80 > 0.75). All sites provided sufficient fine particle organic matter loading for filters [CPOM (suspended)/CPOM (sediment) > 0.5] and stable substrates for Scrapers and Collector-filters (Channel stability > 0.5), excluding S1 and S2. The downstream and inundated sites had normal predator-prey ratios, whereas the CPOM/FPOM ratios were inferior to 0.25 in the whole study area, suggesting a non-functioning riparian zone, except for S2 (CPOM/FPOM = 0.73 > 0.25) (Table 3).

The low CPOM/FPOM ratios seen in the remaining sites implied such a low abundance of Shredders. In the whole study area, the P/P ratio remained less than 0.15, indicating a normal predator-prey interaction. The low CPOM/FPOM ratios seen in other sites implied such a low abundance of Shredders.

### Relationship between the functional structure of aquatic community and environmental factors

**Figure 5.**
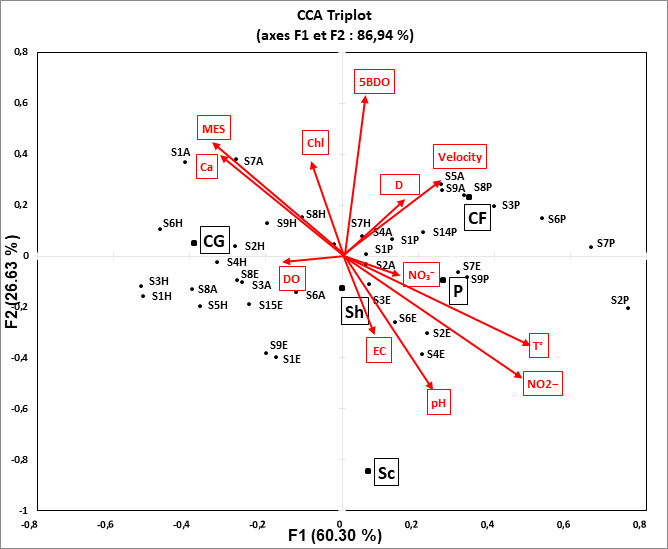
CCA plot depicting relationship between sampling sites. FFGs black squares and measured environmental variables red squares. (FFGs: Collector-gatherers **CG**. Collector-filters **CF**. Shredders **Sh**. Scrapers **Sc** and Predators **P**).

The present Canonical Correspondence Analysis related FFGs dataset to the environmental variables, revealed that the first two axes carried the majority of the total inertia. The CCA allowed us to find out that most of the total inertia is represented by the first axis with the second axis; we obtained 86.94% of the total inertia. This means that the illustration of CCA in only two dimensions (F1 and F2) is largely sufficient to analyse the relationships between sites, FFG categories and environmental variables. The graphical representation of the CCA (Fig. 5) allowed us to visualise simultaneously the objects (in our case, FFGs), sites and abiotic variables. The CCA measured 12 environmental parameters that were strongly linked to the functional guilds designated for this study. Depth, Cl-, BOD_5_, DO, NO_3_-, temperature and salinity were found to have meaningful relationships with FFG groups. The eigenvalues measured the quantity of variation retained by each principal component. With values of 0.10 and 0.04, the first two CCA axes explained 60.30% and 26.63%, respectively (Table 4).

The groups of Predators, Scrapers and Shredders are positively correlated to temperature, NO_2_- and pH in relatively shallow and low-flow sites with acceptable water quality (S6 and S8). Predators, in particular, were shown to present a strong positive association with the nutrients load of NO_2_- and temperature.

## Discussion

### Physicochemical and hydromorphological parameters

Dissolved oxygen, temperature, water velocity, river depth and width, food supplies and land-cover attributes are generally responsible for determining macroinvertebrate assemblages (Lamouroux and Dolédec 2004, Al-Shami et al. 2013). In general, most of the physicochemical parameters were within the accepted guideline averages (WHO 2004, Addo-Bediako 2021). TDS concentrations and conductivity scores increased from upstream to downstream sites, while DO concentrations decreased from upstream to downstream. This observed trend could be the result of induced impacts by human practices, such as agriculture and the catchment of sources in the river midstream going downstream. The degradation of water quality in the Laou watershed is essentially related to the alterations caused by nitrates, phosphates and, to a lesser degree, to the oxidable organic matter in terms of ammonium (Errochdi et al. 2012). The results obtained in this study showed that nutrient levels (NO_3_- and NO_2_-) were quite high at S5, S6 and S9. The high nitrate concentration was caused by leaching or runoff from nearby cultivated land (Modley et al. 2020). Total nutrient content did not change substantially between sites, but it was a major discriminator in the PCA. The physicochemical parameter results revealed a relative fluctuation between sampling sites, while pH remained stable over the sampling period. However, streams that drain agricultural and rural catchments are discriminated by their high conductivity, nutrient load and TDS levels. The flow reduction during the dry season contributed to the seasonal variation in physicochemical conditions, which may adversely impact aquatic assemblages. For example, during the summer, we registered the highest DO and electrical conductivity in S1, although the water temperature was not significantly higher than the other sampling points. These findings imply that anthropogenic variables (hydro-morphological alterations and organic pollution) influence the functional structure of aquatic insects in addition to natural factors. The seasonal dynamic of FFG composition was more pronounced under anthropogenic disturbance, which is manifested by a reduction in the abundance of sensitive groups (Shredders and Scrapers) at the expense of an increase in the abundance of tolerant trophic profiles (Collectors), an observation that was supported by Rimcheska et al. (2022). Collector-filters group was positively correlated to BOD_5_ and current velocity of prospected sites and negatively correlated to pH and conductivity, while the Collector-gatherers category was positively associated with ions like Cl-, Ca^2+^ and MES. Indeed, the CCA model demonstrated that, when water quality improved, the presence of certain FFGs (i.e. Scrapers, Shredders and Predators) increased steadily.

### Functional structure of the aquatic community

The functional structure of aquatic insects within sampling sites seems to be considerably influenced by the variation in environmental parameters and habitat quality attributes. In this study, Collector-gatherers, Predators and Collector-filters outnumbered other specialist trophic groups (Scrapers and Shredders) in terms of their respective abundances. Collector-filters were numerically well-represented amongst the selected rivers, which might be attributed to their capacity to graze on a diverse range of food sources in the water column (Merritt et al. 2002). Collector-gatherers are usually reported as the most numerous functional guilds in tropical and temperate streams (Masese et al. 2014, Mangadze et al. 2019, Lubanga et al. 2021). Collector-gatherers were the most common group in the entire study area and were reported with an important proportion in S1 and S6. Moreover, most collectors are generalist feeders (they collect a wide variety of foods) and may live and develop in a variety of stream bottom environments, enhancing their chances of survival and reproduction. They were crucial in repackaging FPOM into larger particles after ingesting it. In particular, Collector-gatherers increased significantly with the growing pollution rate from rural disturbance, whereas collector-filterers exhibited the opposite pattern (Bere et al. 2016). Thus, the presence and abundance of trophic guild components and functional feeding groups are determined by the availability of certain food supplies (Carrasco-Badajoz et al. 2022, Cummins et al. 2022). However, a higher predator population is linked to a greater availability of prey species (collectors) within the ecosystem. Despite the proportion of prey, the relative abundance of Predators remains restricted to S3 and S8. This finding is consistent with the results of (Fu et al. 2016, Mangadze et al. 2019, Nahli et al. 2023). These authors claimed that the presence of predators and Shredders is considerably lower in highly disturbed streams. Furthermore, the loss of riparian coverage and hydromorphological alteration in S3, which has been partially channeled in several sections, favored the development of r-selected taxa such as collectors, adapted to impaired habitats with slow-flow velocity where particles are more abundant (DeBoer et al. 2020, Šumanović et al. 2021). We hypothesis that the decline in the abundance of Scrapers and Collector-filters at sites S1, S2 and S7 is linked to an increase in water particle charge (i.e. TSD mean = 236.75 ppm at S1).

The abundance of collectors would be linked to their capacity to feed on a broad range of food items compared to specialist groups (i.e. Shredders and Scrapers) (Merritt et al. 2002). Shredders were almost absent in S7 and S8 due to non-functioning riparian regions at these locations. Other authors have linked the distribution of Shredders to water temperature and the mineralization process (Ferreira et al. 2012, Masese et al. 2014), which seems to be consistent with the findings of this study. Sites with high water temperatures were frequently associated with low oxygen levels and extensive aquatic flora. Moreover, Shredders play a leading role in the breakdown of large particles of plant materials into smaller pieces that can subsequently be transferred downstream to other stream consumers (Wallace and Webster 1996, Ramírez and Gutiérrez-Fonseca 2014). In general, we refer to Shredders as consumers of coarse particulate organic matter (CPOM) and producers of fine particle organic matter (FPOM). Concurrently, the number of Shredders and Predators have showed considerable correlation with river oxygenation rate and hydromorphological characteristics (i.e. depth and flow velocity). This distribution of Shredders and Predators in deeper, high-flow, well-oxygenated and less-impacted streams (i.e. S4: DO = 8.02 mg/l) confirms their high sensitivity to anthropogenic perturbations, such as river channeling, land use change, nutrient input and organic effluents (Wallace and Merritt 1980, Masese et al. 2014, Mangadze et al. 2019, Eriksen et al. 2021). The CCA results have demonstrated that physicochemical (i.e. T, pH, BOD_5_, Cl- and NO_3_-) and hydromorphological (i.e. current velocity and depth) factors were amongst the key predictors of shifts in the functional structure of aquatic communities during this survey. Physicochemical parameters and nutrient content play a crucial role in structuring freshwater taxa.

### Ecosystem attributes

The chosen environmental attributes are based on previous documented research (Vannote et al. 1980, Wagner 2001, Merritt et al. 2002, Moyo and Richoux 2017). The balance between autotrophy and heterotrophy is arguably the most fundamental ecosystem attribute (Merritt et al. 2002). Furthermore, the P/R ratio indicated a clear preference for heterotrophy over autotrophy, except for S7, which has a slightly higher P/R ratio (P/R = 0.80 > 0.75), reflecting suitable DO levels generated by autotrophic entities (DO = 8.52 mg/l), noting that high DO levels support diverse macroinvertebrate assemblages in wetland systems (Merritt et al. 1996, Stone and Wallace 1998, Wagner 2001). Moreover, the high P/P ratio (< 0.15) found in this research indicated a strong top-down control over the whole length of the study area, except for S1. The high abundance of Predators over the whole longitudinal gradient could be attributed to food availability and lower competition. Due to the availability of allochthonous resources (leaf litter from overhanging vegetation), Collector-gatherers should co-dominate with Shredders in headwaters (upstream stations) on the grounds that Collector-gatherers use fine particulate organic matter (FPOM) produced by Shredders. The total CPOM to FPOM ratio indicated the availability of food supplies for Shredders, except S3, where Scrapers tend to be more abundant. The majority of Shredders were found in upstream sections of the investigated streams, which might be related to the maximum leaf fall biomass in rivers. Our guild concept might include a small number of Shredders due to the progressive shrinking of the riparian interface as a result of human-induced effects. The availability of canopy cover influenced the number of Shredders in a river. Most of aquatic insects feed on allochthonous organic matter, which is less abundant in open (non-shaded) rivers than in shaded streams (Li et al. 2009, Touma et al. 2009). Our findings confirmed that Shredders are disappearing from sites impacted by anthropogenic disturbances (S7 and S8). In addition, the low channel stability ratio received from sites S1 and S2 is due to a shortage of stable surfaces, suggesting that the FPOM is driven by wastewater discharge rather than natural riparian and hydrological dynamics (Merritt et al. 2017, Nahli et al. 2023). Furthermore, the low CPOM/FPOM ratios seen in several sites implied a low abundance of Shredders vs. Collectors, confirming that most of selected sites (excluding S2) have a non-functional riparian zone, since the Shredder population has been reduced. The dominance of heterotrophy over autotrophic production may be related to the significant contamination caused by animal waste (Masese et al. 2014). The high channel stability revealed the availability of appropriate substrates, such as bedrock, boulders, cobbles and large woody debris that offer stable substrates for filter-feeding and scraping groups.

Our findings showed that the trophic profiles of aquatic insects are substantially related to food resource availability. The presence of the dietary supplies and environmental variability can explain the heterogeneity of FFGs at different sampling sites. According to Barbour et al. (1996), specialized feeders, such as Shredders and Scrapers, are considered to be more sensitive to disturbances, whereas generalist group, such as Collector-gatherers and Collector-filters, are expected to be more tolerant to pollution that may affect resource consumption and habitat use. The dominance of Collectors over the large scale of a river has also been noted in Kenyan highland streams (Masese et al. 2014, Sitati et al. 2021). Overall, the FFG ratios identified a broad human effect, such as vegetation clearing, animal grazing and crop production (Makaka et al. 2018). Although, these findings suggested that functional analysis of FFGs in aquatic communities could be used to survey heavily impacted sites and how profoundly Rifian watercourses have been altered as a result of persistent anthropogenic impacts. Thus, there was no significant variation in the proportions of trophic groups amongst all sites over seasons. This may be explained, in part, by the difficulty of predicting the responses of trophic traits to stressors or by the fact that changes in functional habits were possibly governed by natural driver, such as stream orders, stream width or biotic interactions (Statzner and Beche 2010, Martini et al. 2021).

### Relationship between the functional structure of aquatic insects and environmental factors

As indicated by Galbrand et al. (2007), the modification in trophic structure is frequently symptomatic of a community responding to a particular food supply or to a disturbance regime. According to the CCA results, physicochemical (i.e. T, pH, MES, BOD5, Cl- and NO_3_-) and hydromorphological (i.e. current velocity and depth) factors were amongst the principal predictors for altering the functional structure of aquatic biota. Predators, in particular, were shown to present a strong association to NO_2_- and temperature in deeper, high-flow, well-oxygenated and less-impacted sites, confirming their sensitivity to disturbance. This finding might explain why Collector-gatherers were positively associated with ions load such as Cl-, Ca^2+^ and MES due to their important resistance to nutrient infestation, compared with specialized feeding groups that have constrained trophic niches, such as Collector-filters that required ordinary flow conditions to filter food particles from the water column and, as a consequence, their abundance declined in S2 (V = 0.61 m/s).

## Conclusion

In summary, specialized feeders, such as Shredders and Scrapers, are thought to be more sensitive to disturbances, whereas generalist groups, such as Collector-gatherers and Collector-filters, are considered to be more tolerant to anthropogenic stressors. The changes in FFG composition might serve as a valuable indication of ecosystem variability and recovery after disturbances. Furthermore, the current study lays the groundwork for long-term biomonitoring for management goals. It is anticipated that a comprehensive investigation will be required using the lowest taxonomic level (genus or species) because some families are quite diverse and species within a family certainly belong to various feeding groups. We are completely aware of the limitations of this work, which are frequent in studies that use datasets taken from a public database or literature. Thus, our results should be useful in defining new criteria for measuring the integrity of freshwater ecosystems, as well as in evaluating and forecasting future changes in aquatic communities exposed to human-induced alterations.

## Supplementary Material

50972DE3-C34A-5136-827C-4B218E476943 10.3897/BDJ.11.e104218.suppl1 8046275 Supplementary material 1 Total abundance of captured taxa in the studied sites Data type Occurrences Data File: oo_863124.xlsx https://binary.pensoft.net/file/863124 Sara El Yaagoubi1,*, Majida EL Alami1, Rihab Harrak1, Ahlame Azmizem1, Mohamed Ikssi1, Mohammed Reda Aoulad Mansour2

37F9794B-9956-5221-ADDE-8E6FB071FD3C 10.3897/BDJ.11.e104218.suppl2 Supplementary material 2 Environmental variables (Mean ± SD) measured in the nine sites of the study area Data type Environmental parameters File: oo_863125.xlsx https://binary.pensoft.net/file/863125 Sara El Yaagoubi1, Majida EL Alami1, Rihab Harrak1, Ahlame Azmizem1, Mohamed Ikssi1, Mohammed Reda Aoulad Mansour2

## Figures and Tables

**Table 1.** Calculated ratios of the FFGs used as surrogates of ecosystem function (Cummins et al. 2005).

**Ecosystem attributes**	**Symbols**	**Functional feeding group ratios for attributes**	**General criteria ratio levels**
Autotrophy to heterotrophy index	P/R	Scrapers to Shredders + total Collectors	Autotrophic > 0.75
Predator-prey ratio	P/P	Predators to the total of all other functional groups	< 0.15 indicates a normal predator/prey ratio
Coarse particulate organic matter (CPOM) to fine particulate organic matter (FPOM) index	CPOM/FPOM	Shredders to total collectors	Normal shredder association linked to functioning riparian zone > 0.25
FPOM in transport (suspended) to FPOM storage in sediments	TFPOM/BFPOM	Collector-filters to Collector-gatherers	FPOM transport (in suspension) enriched unusual particulate loading) > 0.50
Substrate (Channel) stability	Channel Stability	Scrapers + Collector-filters to Shredders + Collector-gatherers	Stable substrates (e.g. cobbles, boulders, large woody debris, rooted vascular plants) plentiful > 0.50

**Table 2.** Abundance of functional feeding groups (FFGs) along investigated sites.

**FFGs**	**CG**	**P**	**CF**	**Sh**	**Sc**	**P/Sc**	**CG/Sc**
**S1**	627	169	122	20	54	0	8
**S2**	66	204	15	39	14	0	25
**S3**	172	92	61	16	42	0	2
**S4**	253	283	219	90	44	8	25
**S5**	153	123	133	9	1	2	0
**S6**	353	215	86	23	25	0	8
**S7**	273	136	346	1	29	0	6
**S8**	111	118	70	2	8	0	1
**S9**	101	163	143	22	5	6	0
**Total**	2109	1503	1195	222	222	16	75
% **Total**	**39.47**	**28.14**	**22.37**	**4.16**	**4.16**	**0.30**	**1.40**

**Table 3.** Means and standard deviations (SD) of the different FFG ratios (**P/R**: Autotrophy/heterotrophy index. **CPOM/FPOM**: coarse particle organic matter (CPOM)/fine particle organic matter (FPOM). **P/P**: Predator/Prey).

	**S1**	**S2**	**S3**	**S4**	**S5**	**S6**	**S7**	**S8**	**S9**
**P/R**	0.12±0.20	0.29±0.18	0.26±0.17	0.63±0.09	0.26±0.01	0.26±0.06	0.80±0.09	0.41±0.05	0.58±0.02
**P/P**	0.31±0.02	0.04±0.01	0.02±0.01	0.05±0.01	0.02±0.02	0.04±0.02	0.03±0.02	0.02±0.02	0.03±0.01
**CPOM/FPOM**	0.02±0.02	0.73±0.76	0.07±0.05	0.18±0.11	0.02±0.02	0.08±0.07	0.001±0.002	0.01±0.02	0.08±0.08
**TFPOM/BFPOM**	0.17±0.12	0.23±0.38	0.46±0.63	1.80±2.22	0.65±0.48	0.62±0.84	1.87±1.52	0.93±1.52	1.64±1.99
**Channel Stability**	0.28±0.19	0.32±0.23	0.60±0.55	1.05±0.83	0.61±0.44	0.52±0.50	2.03±1.64	1.04±1.76	1.14±1.18

**Table 4.** Statistical summary of the CCA analysis.

	**F1**	**F2**	F3	F4
Eigen value	**0.104**	**0.046**	0.017	0.005
Inertia (%)	**60.303**	**26.634**	10.134	2.930
% cumulative	60.303	**86.936**	97.070	100.000
**Total Inertia**	23.485	10.373	3.947	1.141
% cumulative	23.485	33.858	37.805	38.946
